# The Role of Cacao Powder in Enhancing Skin Moisture and Reducing Wrinkles: A 12-Week Clinical Trial and In Vitro Study

**DOI:** 10.3390/cimb46110746

**Published:** 2024-11-06

**Authors:** Sang Gyu Lee, Ngoc Ha Nguyen, Young In Lee, Inhee Jung, In Ah Kim, Hyunsook Jang, Hoyeon Shin, Ju Hee Lee

**Affiliations:** 1 Department of Dermatology & Cutaneous Biology Research Institute, Yonsei University College of Medicine, Seoul 03722, Republic of Korea; dltkdrb5658@yuhs.ac (S.G.L.); ylee1124@yuhs.ac (Y.I.L.); 2 Department of Dermatology, University of Medicine and Pharmacy, Ho Chi Minh City 17000, Vietnam; nguyenngocha7996@gmail.com; 3 Scar Laser and Plastic Surgery Center, Yonsei Cancer Hospital, Seoul 03722, Republic of Korea; 4 Global Medical Research Center Co., Ltd., Seoul 06526, Republic of Korea; ihjung@gmrc.co.kr (I.J.); inah@gmrc.co.kr (I.A.K.); 5 Lotte R&D Center, Seoul 07594, Republic of Korea; hyunsook.jang@lotte.net (H.J.); hoyeon.shin@lotte.net (H.S.)

**Keywords:** cacao powder, skin moisture, skin senescence, wrinkles

## Abstract

Skin aging is driven by a combination of internal and external mechanisms, with ultraviolet (UV) radiation being a prominent external factor contributing to photoaging. Photoaging manifests through several signs, including decreased skin hydration, diminished elasticity, coarse wrinkles, and dyspigmentation. Cacao beans, known for their flavonoids and polyphenols, offer potential anti-aging benefits. To explore this, we conducted a study using both in vitro experiments and clinical trials. Our results demonstrated that cacao powder significantly improved skin hydration and moisture retention in both experimental settings. Specifically, in UVB-damaged human dermal fibroblasts (HDFs) and H_2_O_2_-treated keratinocytes (KCs), cacao powder displayed notable antioxidant properties. Furthermore, cacao powder enhanced the activity of antioxidant enzymes and promoted the production of hyaluronic acid in KCs, contributing to better skin hydration. It also effectively inhibited the expression of matrix metalloproteinase-1, an enzyme associated with wrinkle formation, and stimulated collagen synthesis in HDFs. Clinical trials conducted on participants with aged skin revealed a significant improvement in skin hydration and a reduction in skin wrinkles after 12 weeks of cacao powder consumption, supporting the in vitro findings. These results suggest that cacao powder holds promise as a natural ingredient for improving skin hydration and reducing wrinkles, underscoring its potential in anti-aging skincare.

## 1. Introduction

Skin senescence, which reduces the skin’s natural ability to defend against damage from ultraviolet (UV) radiation, oxidation, and microbes, occurs due to internal and external mechanisms [1]. UV radiation is a major factor in the external mechanism of this phenomenon, causing photoaging [2]. Eventual manifestations of photoaging encompass reduced skin hydration, coarse wrinkles, cutaneous dyspigmentation, and diminished elasticity [3]. Continuous UV radiation exposure limits procollagen production and degrades collagen fibers, partly due to the overproduction of matrix metalloproteinases (MMPs) and the elevated production of reactive oxygen species (ROS) [4]. UVB-induced wrinkle formation is also associated with SERPIN B6, a member of the superfamily of serine protease inhibitors. SERPIN B6 inhibits cathepsin G activity, which may help prevent UVB-induced wrinkling by reducing extracellular matrix damage and the upregulation of MMPs associated with skin photoaging [5]. Skin moisturization, primarily facilitated by hyaluronic acid (HA) molecules found in the epidermis and dermis, is also altered in photoaged skin. Reduced levels of hyaluronan synthases (HAS) were also observed in dermal fibroblasts due to photoaging [6]. HA is synthesized by three hyaluronan synthase isoforms (HAS1, HAS2, and HAS3). In particular, HAS2 is the most highly expressed isoform in keratinocytes. Reduced levels of HAS2 were also observed in aged keratinocytes. Furthermore, UV radiation also affects nuclear factor erythroid 2-related factor 2 (NRF2), a key regulator of enzymes in antioxidant responses, such as glutathione peroxidase (GPx) and superoxide dismutase (SOD) [7]. Reverting these photoaging-derived consequences has been achieved through a number of therapeutic modalities, namely light-based devices, topical formulations, and oral and injectable products [8,9,10].

Cacao beans and their derivatives, such as cacao powder, beverages, and chocolate, have long been used as a source of nourishment or indulgence, possessing numerous beneficial properties. Harvested from the Theobroma cacao tree, cacao contains an abundance of lipids, fibers, minerals, alkaloids like theobromine and caffeine, and most prominently, polyphenols and flavanols, which mainly consist of epicatechins, catechins, and procyanidins [11]. These bioactive phytochemicals promote various physiologically beneficial properties, such as protecting against oxidation, microbes, and inflammation, improving cardiovascular and physical functions, regulating blood glucose, and preventing cancer, diabetes, depression, and photoaging [12]. Specifically, cacao flavanols, a type of flavonoid, known for their antioxidant properties, have been associated with various health benefits, including cardiovascular and cognitive health [13]. Given this, the European Food Safety Authority has recognized the endothelial-dependent vaso-relaxation properties of cacao flavanols as a functional health benefit, applicable across all European Union member states [14,15]. Regarding aging, a few clinical trials have demonstrated the moisturizing, anti-wrinkle, and antioxidant properties of cacao. High-flavanol cacao beverages considerably improved minimal erythema dose, skin moisturization, transepidermal water loss, and skin elasticity and reduced wrinkles and skin roughness in photoaged subjects [16,17]. Mouse model studies showed the protective function of cacao extract against skin wrinkles induced by UVA and UVB radiation, as well as its mitigation against oxidative stress in rat brains [18,19,20]. In vitro testing also demonstrated that cacao powder exhibited anti-wrinkle effects against UVB-induced damage in human dermal fibroblasts (HDFs), while its constituent compound, epicatechin, can shield HDF against UVA-induced cell death [21]. Furthermore, cacao polyphenols extracted from cacao powder exhibited antioxidant and anti-inflammatory properties in cultured human promyelocytic leukemia cells [22]. These results corroborate the potential anti-aging effects of cacao-derived products.

Nonetheless, there is a lack of in vitro studies on skin cells to assess the hydration improvement and antioxidant function of cacao powder. In addition, the effect of cacao on skin moisture is controversial, with one study recording an improvement in skin hydration [17], while others failed to recreate such a result [16,23]. Therefore, we conducted this study to analyze the preventive effects of cacao powder against photoaging and to elucidate the previous controversial findings on skin hydration. The primary objectives are the clinical assessment of skin moisturization and wrinkles in middle-aged subjects with dry, wrinkled skin and the laboratory evaluation of cutaneous hydration as well as wrinkles via human epidermal keratinocytes (KCs) and HDF. Our secondary aim is to analyze the antioxidant property of cacao powder in UVB-irradiated HDFs and H_2_O_2_-treated KCs.

## 2. Materials and Methods

### 2.1. In Vitro Study on Cacao Powder

#### 2.1.1. Test Product

The cacao powder was purchased from Barry Callebaut (Pasir Gudang, Malaysia) and the N-acetyl cysteine (NAC; A9165), quercetin (QC; PHR1488), and retinoic acid (RA; R2625) were purchased from Sigma Aldrich (Saint Louis, MO, USA).

#### 2.1.2. Cell Culture

The HDF cells and KC cells were purchased from Thermo Fisher Scientific (Waltham, MA, USA). The HDF cells were cultured in Dulbecco’s modified Eagle’s medium (DMEM; Lonza, Walkersville, MD, USA) containing 1% penicillin–streptomycin (Gibco, Grand Island, NY, USA) and 10% fetal bovine serum (Gibco), while the KC cells were cultured using EpiLife^TM^ medium with 60 μM calcium (Gibco) and Human Keratinocyte Growth Supplement (Gibco). The incubation was at 37 °C in a humidified atmosphere with 5% CO_2_. All cells used in this study were at passages 5–7.

#### 2.1.3. Cell Cytotoxicity

To determine the cell cytotoxicity of cacao powder dissolved in 50% ethanol, we used the cell counting kit-8 (Dojindo, Mashiki, Japan) assay according to the kit’s instructions. We prepared the cacao powder dissolved in 50% ethanol at a concentration of 5 mg/mL. This solution was subsequently diluted with 1 mg/mL DMEM to achieve a final concentration of 10 µg/mL. We visually confirmed the complete dissolution of the cacao powder prior to conducting the assay. To establish a range of test concentrations, we performed a serial dilution from this initial 10 µg/mL concentration, resulting in the following concentrations: 0.313, 0.625, 1.250, 2.500, 5.000, and 10.000 μg/mL. HDF and KC cells were seeded at a density of 5 × 10^3^ cells per well in a 96-well plate. When the cell density reached approximately 80%, the cells were treated with six different concentrations of cacao powder (0.313, 0.625, 1.250, 2.500, 5.000, and 10.000 μg/mL) and incubated for 24 h. The absorbance was measured at 450 nm using an ELISA microplate reader (VersaMax, Molecular Devices, San Jose, CA, USA).

#### 2.1.4. Improvement Efficacy Assessment

The antioxidant efficacy of cacao powder on KC cells was measured using an OxiTEC^TM^ SOD assay kit (BIOMAX, Gyeonggi-do, South Korea) and GPx assay kit (Abcam, Cambridge, UK). KC cells were seeded onto a 6-well plate at a density of 5 × 10^4^ per well; when the cell density reached approximately 80%, the cells were treated with three concentrations of cacao powder (2.500, 5.000, and 10.000 μg/mL) and incubated for 24 h. Afterward, for induction of oxidant stress, KC cells were treated with 200 μM H_2_O_2_ for 6 h and harvested for protein quantification.

To assess the moisture improvement efficacy of cacao powder on KC cells, we used the Hyaluronan Quantikine enzyme-linked immunosorbent assay (ELISA) kit (R & D Systems, Minneapolis, MN, USA) and the Human HAS-2 ELISA kit (MyBioSource, San Diego, CA, USA). KC cells were seeded in a 6-well plate at a density of 5 × 10^4^ per well. When the cell density reached approximately 80%, the cells were treated with three concentrations of cacao powder, incubated for 24 h, and harvested for protein quantification.

The wrinkle improvement efficacy of cacao powder on HDF cells was measured using the Human Pro-Collagen I α1 SimpleStep ELISA kit (Abcam) and Human MMP-1 ELISA kit (Abcam). HDF cells were seeded in a 6-well plate at a density of 5 × 10^4^ cells per well, and when the cell density reached approximately 80%, cells were treated with three concentrations of cacao powder, incubated for 24 h, irradiated with 30 mJ/cm^2^ UVB, and then incubated for 24 h. The HDF cells and supernatants were harvested, and protein quantification was performed using the Bicinchoninic Acid Proteins kit (Sigma-Aldrich) or using all of the standard solutions enclosed in each kit to quantify the protein. Optical densities were measured using a microplate reader (VersaMax). As a positive control, 2.5 mM NAC (dissolved in PBS), 12.5 μM QC (dissolved in ethanol), and 1.25 μM RA (dissolved in ethanol) were used to assess the antioxidant efficacy, moisture improvement, and wrinkle improvement, respectively.

#### 2.1.5. RNA Extraction and qRT-PCR

HDF cells were seeded in a 6-well plate at a density of 5 × 10^4^ cells per well; when the cell density reached approximately 80%, cells were treated with three concentrations of cacao powder and incubated for 24 h. The TRIzol reagent (Invitrogen, Waltham, MA, USA) and RNA to cDNA EcoDry^TM^ Premix kit (Clontech, Takara Sake, Berkley, CA, USA) were used for the extraction of total RNA and synthesis of cDNA. Quantitative real-time reverse transcription polymerase chain reaction (qRT-PCR) was conducted using the synthesized cDNA, the Taqman primer (NRF2, Hs00975961_g1; SERPIN B6, Hs01063501_m1; Applied Biosystems), the Taqman Gene Expression Master Mix (Applied Biosystems, Waltham, MA, USA), and the QuantStudio 3 RealTime Polymerase Chain Reaction System (Applied Biosystems). Comparison of the mRNA expression levels was normalized to GAPDH (Hs02786624_g1, Applied Biosystems) as a housekeeping gene and calculated through the 2^−ΔΔCt^ method. As a positive control, 1.25 μM RA was used in this experiment.

### 2.2. Clinical Efficacy of Cacao Powder on Skin Rejuvenation

#### 2.2.1. Test Product and Participants

The test product (containing 4 g cacao powder, Lotte R&D Center, Seoul, Republic of Korea) was in pill form with cacao powder as the major ingredient, to be taken with water at 4 g/day. To reach the total intake of 4 g of cacao powder per day, participants consumed 97 pills, all contained in a single packet, totaling 4.2 g when including other ingredients. The cacao powder used in this study contained 71.5 mg/g of flavanol and the test group consumed 286 mg of total flavanols per day. The placebo product (placebo pill) was made using rice powder and had the same shape, color, and weight as the test product. The ingredients of each pill are listed in Table S1.

The participants of this clinical study were aged 35 to 60 years and had dry skin and wrinkles around the eyes. The clinical study method, study period, expected effects of the products, and possible side effects were thoroughly explained to all participants before they gave their consent. A total of 100 participants were recruited in consideration of a dropout rate of 20%.

#### 2.2.2. Clinical Study Design

The study was conducted in accordance with the Declaration of Helsinki and approved by the Global Medical Research Center’s Clinical Trial Review Board (IRB review number: GIRB-22N15-KY). This clinical trial was designed as a randomized, double-blind, placebo-controlled, parallel trial. Participants who voluntarily signed the consent form underwent various assessments, including demographic investigations, medical and drug administration history, lifestyle, physical examinations and measurements (height, weight), vital signs (blood pressure, pulse), clinicopathological examinations, a pregnancy test (for women of childbearing age), cosmetic usage analyses, and skin moisture measurements. Participants were randomly allocated to either the placebo group or the test group. Participants consumed the test products or placebo products for a total of 12 weeks, with an allocation ratio of 1:1. Registered participants visited the institute at weeks 0, 6, and 12 for skin moisture tests. Participants who suffered adverse reactions after taking the test product were asked to report them during the clinical study.

#### 2.2.3. Clinical Improvement Efficacy Assessment

To accurately assess the improvement of skin wrinkles, a dermatologist evaluated the skin grade according to predefined criteria (Table S2). Participants who received a grade of 4 or higher were further evaluated for clinical improvement efficacy.

To assess the skin moisture content after intake of the test products, we used Corneometer CM825 (Courage + Khazaka Electronic GmbH, Cologne, Germany) on the participant’s skin surface. Measurements were conducted at a temperature of 20–24 °C and humidity of 40–60%, after washing the face and resting for 30 min, with water intake restricted for 1 h before measurement. The measurement areas were the left and right eye corners and the tip of the nose. All measurements were repeated more than three times, and results were compared between baseline, after 6 weeks, and after 12 weeks of product intake. Participants with skin moisture below 49 A.U. were selected for this assessment.

The assessment of the periorbital skin wrinkles was conducted using phase-shift rapid in vivo measurement of skin (PRIMOS, PRIMOS CR, SnT Lab, Seoul, Republic of Korea) at baseline and after taking the product for 6 and 12 weeks. Among the PRIMOS’ skin surface measurement parameters, the periorbital skin wrinkles were measured using Rmax and Rp on the right side of the face.

#### 2.2.4. Data Statistics

All data were presented as mean ± standard deviation. Baseline, 6 weeks, and 12 weeks’ clinical parameters were compared with each other using two sample *t*-tests or the Wilcoxon rank sum test based on the normality test results (placebo group; *n* = 36, test group; *n* = 39). The resulting data obtained from the in vitro study were analyzed using the independent samples *t*-test. Statistical analyses were conducted using SAS^®^ (Version 9.4, SAS Institute, Cary, NC, USA) and statistical significance was determined at *p* < 0.05, *p* < 0.01, and *p* < 0.005. Each experiment in this study was conducted at least three times (*n* ≥ 3), and the resulting data were utilized for statistical analysis. The error bars are based on standard deviation.

## 3. Results

### 3.1. In Vitro Study on Cacao Powder

#### 3.1.1. Cellular Effect on the Expression Levels of Hyaluronic Acid and HAS2

**Figure 1:**
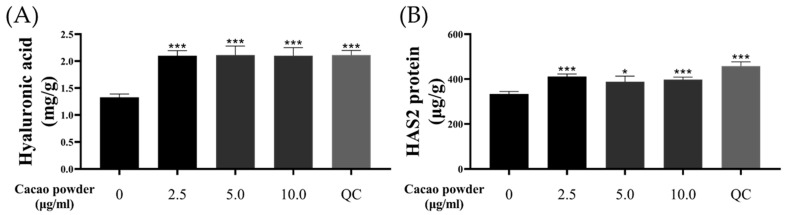
Effects of cacao powder on moisture improvement. The treatment of cacao powder significantly induced the synthesis and production of hyaluronic acid (**A**) and HAS2 protein (**B**) on KC. KC, human epidermal keratinocyte; QC, quercetin, * *p* < 0.05, *** *p* < 0.005, independent samples *t*-test. *n* = 3, The error bars are based on standard deviation.

The results of the cell cytotoxicity assay demonstrated that cacao powder is safe (Figure S1). Based on these findings, we selected three concentrations out of the six tested for use in subsequent experiments. The production levels of HA and HAS2 were measured in KC cells to assess moisture improvement. The control group was not exposed to any stresses or treatments. QC at 12.5 μM was used as a positive control. Treatment with 2.500, 5.000, and 10.000 μg/mL of cacao powder significantly increased the production of HA compared to the control group, with results comparable to the QC treatment group (control; 1.329 ± 0.059 mg/g, 2.500 μg/mL; 2.100 ± 0.095 mg/g, 5.000 μg/mL; 2.113 ± 0.167 mg/g, 10.000 μg/mL; 2.101 ± 0.151 mg/g, QC; 2.113 ± 0.085 mg/g, Figure 1A, *** *p* < 0.005). Similarly, HAS2 protein production was enhanced by treatment with the three concentrations of cacao powder and QC compared to the control group (control; 333.530 ± 11.262 μg/g, 2.500 μg/mL; 411.173 ± 11.522 μg/g, 5.000 μg/mL; 387.954 ± 25.092 μg/g, 10.000 μg/mL; 397.027 ± 11.915 μg/g, QC; 457.390 ± 19.294 μg/g, Figure 1B, * *p* < 0.05, *** *p* < 0.005). Therefore, cacao powder treatment in KC cells improved moisture levels, with effects similar to those of QC, the positive control.

#### 3.1.2. In Vitro Molecular Experiments on Wrinkle Improvement Effect

**Figure 2:**
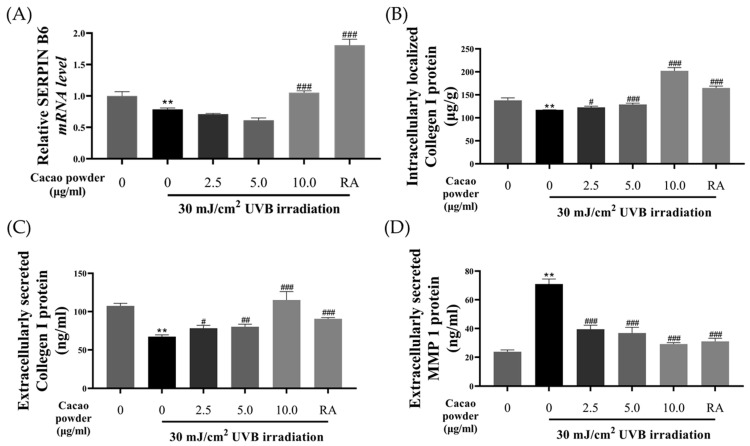
Wrinkle improvement effects of cacao powder. The treatment of cacao powder significantly induced the *SERPIN B6* gene expression reduced by 30 mJ/cm^2^ UVB irradiation on HDF (**A**). The treatment of cacao powder showed the effects of collagen type I protein synthesis, both intracellular (**B**) and extracellular (**C**), more than positive control on HDF. The increased MMP1 protein was reduced by the treatment of cacao powder (**D**). HDF, human dermal fibroblast; UVB, ultraviolet B; MMP1, matrix metalloproteinase-1; RA, retinoic acid, ** *p* < 0.01, compared to the control, # *p* < 0.05, ## *p* < 0.01, ### *p* < 0.005 compared to UVB treatment group, independent samples *t*-test. *n* = 3, The error bars are based on standard deviation.

The effects of wrinkle improvement were investigated using PCR and ELISA. The control group was not exposed to any stresses or treatments. RA at 1.25 μM was used as a positive control. UVB irradiation at 30 mJ/cm^2^ on HDF cells significantly inhibited *SERPIN B6* gene expression compared to the control group (control; 1.000 ± 0.070, UVB irradiation; 0.788 ± 0.020, Figure 2A, ** *p* < 0.01 compared to the control). As a result, the treatment of 10.000 μg/mL cacao powder or RA significantly induced *SERPIN B6* gene expression (10.000 μg/mL; 1.055 ± 0.027, RA; 1.807 ± 0.095, Figure 2A, ### *p* < 0.005 compared to UVB irradiation group).

Moreover, we investigated the effect of cacao powder on the secretion and expression of collagen type I protein (Figure 2B,C). The intracellular concentration of collagen type I protein was reduced through the UVB irradiation (control; 138.025 ± 5.319 μg/g, UVB irradiation; 117.437 ± 0.241 μg/g, Figure 2B, *** *p* < 0.005 compared to the control). In contrast, treatment with 2.500, 5.000, and 10.000 μg/mL of cacao powder significantly increased the collagen type I protein concentration compared to the UVB irradiation group (2.500 μg/mL; 122.713 ± 2.592 μg/g, 5.000 μg/mL; 129.007 ± 2.983 μg/g, 10.000 μg/mL; 202.186 ± 7.003 μg/g, Figure 2B, # *p* < 0.05, ### *p* < 0.005 compared to UVB irradiation group). Notably, treatment with 10.000 μg/mL of cacao powder led to a significantly higher concentration than the RA treatment group (RA; 164.820 ± 4.183 μg/g, Figure 2B, ### *p* < 0.005 compared to the UVB irradiation group). Similarly, the results for the extracellular concentration of collagen type I protein mirrored the intracellular results (control; 107.426 ± 3.440 ng/mL, UVB irradiation; 67.295 ± 2.419 ng/mL, 2.500 μg/mL; 78.307 ± 3.748 ng/mL, 5.000 μg/mL; 80.202 ± 3.419 ng/mL, 10.000 μg/mL; 115.116 ± 11.038 ng/mL, RA; 90.544 ± 1.594 ng/mL Figure 2C, *** *p* < 0.005 compared with control group, # *p* < 0.05, ### *p* < 0.005 compared with UVB irradiation group).

The MMP1 protein, which degrades collagen type I, was also measured in HDF cells (Figure 2D). UVB irradiation significantly increased MMP1 protein production compared to the control (control; 23.853 ± 1.268 ng/mL, UVB irradiation; 70.989 ± 3.521 ng/mL, Figure 2D, *** *p* < 0.05 compared with the control). Treatment with cacao powder inhibited the UVB-induced increase in MMP1 protein production, with the 10.000 μg/mL treatment showing a greater inhibitory effect than the RA treatment (2.500 μg/mL; 39.490 ± 2.839 ng/mL, 5.000 μg/mL; 36.831 ± 3.981 ng/mL, 10.000 μg/mL; 29.159 ± 1.019 ng/mL RA; 90.544 ± 1.594 ng/mL, Figure 2D, ### *p* < 0.005 compared to UVB irradiation).

#### 3.1.3. Evaluation of Antioxidant Effects

**Figure 3:**
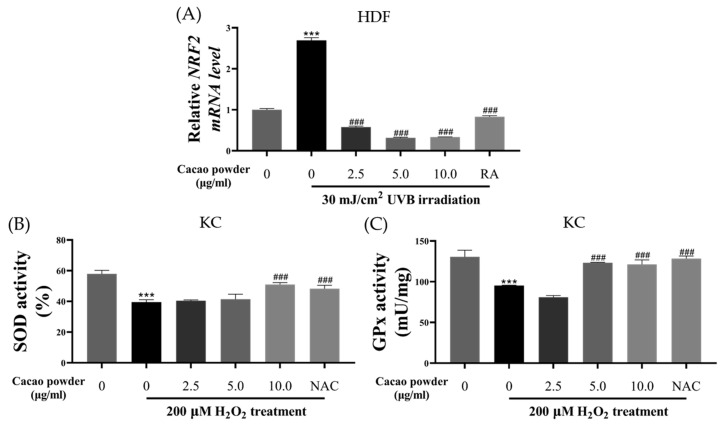
Antioxidant effects of cacao powder. The treatment of cacao powder significantly reduced the *NRF2* gene expression induced by 30 mJ/cm^2^ UVB irradiation on HDF (**A**). The SOD activity (**B**) and GPx activity (**C**) were upregulated by the treatment of cacao powder on KC, which was similar to the positive control group. HDF, human dermal fibroblast; KC, human epidermal keratinocyte; UVB, ultraviolet B; NRF2, nuclear factor erythroid 2-related factor 2; SOD, superoxide dismutase; GPx, glutathione peroxidase; RA, retinoic acid, *** *p* < 0.005, compared to the control, ### *p* < 0.005 compared to UVB treatment group or NAC treatment group, independent samples *t*-test. *n* = 3, the error bars are based on standard deviation.

To evaluate the antioxidant effects of cacao powder, we assessed *NRF2* gene expression levels in HDF cells, as well as GPx and SOD activity in KC cells (Figure 3). The control group was not exposed to any stresses or treatments. RA at 1.25 μM and NAC at 2.5 mM were used as positive controls. The *NRF2* gene expression level in the control group was 1.000 ± 0.031, while UVB irradiation significantly increased to 2.693 ± 0.065 (Figure 3A, *** *p* < 0.005 compared to the control). In contrast, for the treatments of 2.500, 5.000, and 10.000 μg/mL cacao powder, the expression levels of the *NRF2* gene were 0.315 ± 0.017, 0.336 ± 0.005, and 0.831 ± 0.026, respectively (Figure 3A, ### *p* < 0.005 compared to the UVB irradiation). The RA treatment also decreased *NRF2* gene expression, but the levels with the cacao powder treatment were lower than with RA (0.831 ± 0.026, Figure 3A, ### *p* < 0.005 compared to the UVB irradiation).

SOD activity in the control was 57.964 ± 2.245%, while treatment with 200 μM H_2_O_2_ reduced it to 39.563 ± 1.556% (Figure 3B, *** *p* < 0.005 compared to the control). Among the treatment groups, 10.000 μg/mL of cacao powder resulted in the highest SOD activity, surpassing even the positive control (2.500 μg/mL; 40.426 ± 0.527%, 5.000 μg/mL; 41.403 ± 3.279%, 10.000 μg/mL; 51.006 ± 1.208%, NAC; 48.304 ± 2.184%, Figure 3B, ### *p* < 0.005 compared to H_2_O_2_ treatment).

Similarly, the results of the GPx activity were reduced more by the H_2_O_2_ treatment compared to the control (control; 130.564 ± 8.097 mU/mg, H_2_O_2_ treatment; 95.316 ± 0.444 mU/mg, Figure 3C, *** *p* < 0.005 compared to the control). Treatment with 5.000 and 10.000 μg/mL of cacao powder significantly increased GPx activity compared to the H_2_O_2_ group, with levels comparable to the NAC treatment (2.500 μg/mL; 80.925 ± 2.131 mU/mg, 5.000 μg/mL; 123.303 ± 0.520 mU/mg, 10.000 μg/mL; 121.367 ± 5.421 mU/mg, NAC; 128.404 ± 3.120 mU/mg, Figure 3C, ### *p* < 0.005 compared to H_2_O_2_ treatment).

### 3.2. Clinical Efficacy of Cacao Powder on Skin Rejuvenation

#### 3.2.1. Participants Characteristics

The clinical study included a total of 100 participants, who were evenly divided into a placebo group and a test group. Among them, 21 participants dropped out due to scheduling conflicts. The recruitment, screening, and randomization process for participants is illustrated in Figure S2. No adverse reactions were reported from the intake of either the test products or the placebo. After 12 weeks, clinical data from 75 participants were available for final analysis (placebo group: 36 participants; test group: 39 participants). There were no significant differences in demographic and lifestyle factors, such as age, gender, alcohol consumption, smoking habit, outdoor activity time, and sunscreen use, between the two groups (Table S3). Regarding nutrition, our participants followed a consistent diet throughout the study and there were no notable differences between the two groups (Table S4).

#### 3.2.2. Clinical Improvement Efficacy of Cacao Powder Intake

**Figure 4:**
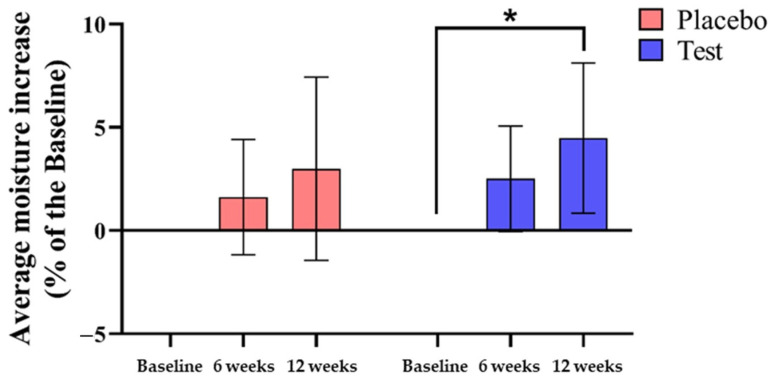
Skin moisture improvement from cacao powder intake. The skin moisture was increased by taking the test products for 12 weeks compared to the baseline. Placebo, placebo products intake; Test, test products intake; * *p* < 0.05, two sample *t*-test. Placebo group; *n* = 36, Test group; *n* = 39. The error bars are based on standard deviation.

After evaluating the participants’ skin grades, 39 participants in the test group and 36 participants in the placebo group had a skin grade of 4 or higher, qualified for investigation for changes in skin moisture, and had periorbital skin wrinkles. Also, the participants who had a skin grade of 4 or higher had an average age of 47.87 ± 6.89. All participants took either the test products or the placebo products once a day with water for 12 weeks. Moisture content on the face was measured, and values were compared to baseline levels. In the placebo group, the moisture content increased by 1.62 ± 2.80% after 6 weeks and by 2.99 ± 4.44% after 12 weeks. In the test group, moisture content increased by 2.52 ± 2.56% after 6 weeks and by 4.48 ± 3.64% after 12 weeks. The results after 12 weeks in the test group showed a significant increase from baseline (baseline; 43.956 ± 3.602 A.U., 12 weeks; 45.882 ± 3.550, two-sample *t*-test, Figure 4, * *p* < 0.05).

**Figure 5:**
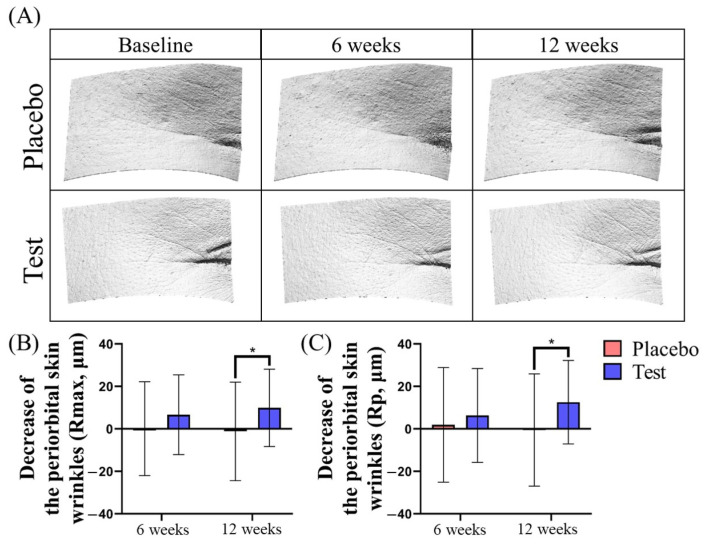
The periorbital skin wrinkles’ improvement from cacao powder intake. The periorbital skin wrinkles’ improvement was visualized (**A**). The moisture level of the periorbital skin wrinkles increased by taking the test products for 12 weeks compared to the baseline (**B**,**C**). Placebo, placebo products intake; Test, test products intake; * *p* < 0.05, independent samples *t*-test. Placebo group; *n* = 36, Test group; *n* = 39. The error bars are based on standard deviation.

The change in periorbital skin wrinkles after the test and placebo products were consumed is visually depicted in Figure 5A. The Rmax value changes for periorbital wrinkles in the placebo group were −0.31 ± 22.11 after 6 weeks and 1.14 ± 23.20 after 12 weeks, while in the test group, the changes were −6.66 ± 18.76 after 6 weeks and −9.92 ± 18.19 after 12 weeks (Figure 5B). After 12 weeks, a significant difference in the change between the placebo and test groups was observed (* *p* < 0.05). A similar trend was observed in the Rp values (placebo 6 weeks: −1.83 ± 27.01, placebo 12 weeks: 0.55 ± 26.44; test 6 weeks: −6.31 ± 22.09, test 12 weeks: −12.57 ± 19.65, Figure 5C, * *p* < 0.05).

## 4. Discussion

Cacao beans possess a plethora of flavanols and polyphenols that are beneficial for human health, including the integumentary system [11]. Our study aimed to evaluate the anti-aging capabilities of cacao powder based on three criteria: skin hydration, antioxidation, and wrinkle prevention. Furthermore, our study involved participants with an average age of 47.87 ± 6.89 years and included a 12-week course of cacao powder supplementation, limiting the impact of seasonal fluctuations in skin hydration status. Our results showed that cacao powder enhanced skin moisturization and reduced skin wrinkles, both clinically and in vitro via HDF and KC. Additionally, cacao powder also demonstrated its antioxidant properties via UVB-irradiated HDF and H_2_O_2_-treated KC. To the best of our knowledge, our study was the first to assess the moisturization and antioxidant abilities of cacao powder on HDF and KC in vitro.

Regarding the skin hydration enhancement of cacao powder, our laboratory results showed an increase in HA and HAS-2 in KC treated with cacao powder. In terms of clinical cutaneous moisture, the test group demonstrated better improvement than the placebo group after a 12-week consumption of cacao powder containing 286 mg of flavanol. HA, a primary constituent of the extracellular matrix (ECM) of the skin, can retain water and aid in structural support [24]. HA is synthesized by HASs, which are membrane-bound enzymes on the plasma membrane and have three isozymes: HAS-1, 2, and 3 [24]. Aging decreases the production of dermal HA, leading to water volume loss and dry skin. To counter this, cacao can concurrently ameliorate transepidermal water loss and improve cutaneous microcirculation [17,25]. Enhanced dermal blood flow influences the regulation of temperature, nutrients, and oxygen supply, ultimately deciding the condition of the skin [26]. Our results in terms of skin hydration were consistent with the first study that evaluated the anti-photoaging properties of cacao, in which daily consumption of cacao powder containing 326 mg of flavanol increased skin moisture after 12 weeks [17]. However, other evidence for this aspect of improvement is still controversial. Yoon et al. reported that a 24-week daily consumption of 320 mg of cacao flavanol did not significantly improve skin moisture, which could be attributed to the majority of participants finishing the study in winter when skin hydration was suboptimal compared to other seasons [16]. Another study carried out by Mogollon et al. used high-flavanol chocolate containing 600 mg of flavanol and also failed to reproduce these results [23]. However, the author noted that the participants joined the study in different seasons, which led to variations in the results [23]. Collectively, our results suggest that cacao powder can strengthen the water-retaining capability of the skin, but more research is warranted to ascertain the optimal dose of flavanols to achieve this enhancement.

Our study utilized UVB radiation, which can penetrate the upper dermis layer of the skin, to simulate photoaging in HDFs due to their lower quality of genome repair than KCs [27]. Our tests presented that exposure to UVB radiation reduced the expression of intracellular and extracellular collagen type I as well as increased MMP-1 expression. Notably, pre-treatment with cacao powder was able to prevent these effects in UVB-irradiated HDF. In addition, we observed that with increasing concentrations of cacao powder, the expression of intracellular and extracellular collagen manifested an upward trend, while MMP-1 had an opposite effect. This suggests that higher titers of cacao powder can elicit a better reduction in skin wrinkles. Our laboratory results were further strengthened by our clinical observations, in which we recorded a better improvement in skin wrinkles from the baseline in the test group than the placebo group. On a molecular basis, the formation of wrinkles is inextricably linked to the degradation of extracellular collagen type I and diminished production of intracellular collagen type I, or procollagen. These dermal regressions originate from UV radiation stimulating ROS-induced MMP-1 production and inhibiting transforming growth factor-beta signaling, respectively [4]. Our outcomes were in line with other studies. A mouse skin model study conducted by Kim et al. showed that cacao powder taken orally decreased the formation of wrinkles and collagen loss in UV-irradiated areas [20]. The authors also reported that cacao powder inhibited the UV-induced upregulation of MMP-1 in HDFs [20]. In humans, a 24-week supplementation of cacao as a beverage improved the appearance of wrinkles and skin elasticity [16]. The wrinkle regression efficacy of cacao powder is comparable to that of other widely utilized anti-wrinkle preparations. Oral HA intake has shown similar wrinkle-reducing effectiveness in many studies with the same duration as our study [28,29]. Low- and high-molecular HA-containing topical agents also exhibited anti-wrinkle properties, with a superior effect for the former [30]. For collagen, an abundance of evidence exists to substantiate its ability to treat skin wrinkles, either in a topical or oral formulation [31]. Nevertheless, the variety of wrinkle assessment tools in these studies makes a direct comparison with cacao powder difficult, necessitating future studies on this matter. Other phytochemical-derived topical formulations, such as *Ginkgo biloba*, *Camellia sinensis*, *Aspalathus linearis*, and *Glycine soja*, also showed promising results [32]. From these findings, our study contributes more clinical and molecular evidence to the anti-wrinkle ability of cacao powder.

Another pair of molecules that are possibly related to the formation of wrinkles is cathepsin G and SERPIN B6. Cathepsin G is a lysis enzyme of fibronectin in the ECM which can mediate the expression of MMPs [33]. Meanwhile, SERPIN B6 is an inhibitor of cathepsin G secreted by cells in order to counter ectopic activation of cathepsin G during inflammation, thereby limiting the activation of MMPs [5]. Interestingly, our study observed a decreased expression of the *SERPIN B6* gene in HDFs irradiated by UVB, as well as an increased expression of MMP-1 as previously presented. In contrast, cacao powder pretreatment was able to upregulate *SERPIN B6* genes and reduce MMP-1 expression, specifically at a 10.000 µg/mL concentration. Kim et al. found that oral intake of cacao powder decreased cathepsin G expression and increased SERPIN B6 expression in the ECM of mouse skin, as shown by genetic analysis [20]. Our similarity with these findings may suggest a possible pathway through which cacao powder can alleviate the appearance of wrinkles. This pathway could specifically be the activation of SERPIN B6 to reduce cathepsin G-induced MMP-1. More studies are warranted to ascertain the relationship between SERPIN B6, cacao powder, and skin wrinkles.

ROS-induced damage in photoaging is a common pathway in the formation of skin wrinkles and reduced cutaneous hydration. As previously mentioned, UV-induced ROS production leads to the enhanced activation of MMP-1 through AP-1, thereby promoting the degradation of collagen type I and subsequently creating wrinkles [4]. On the other hand, ROS also negatively influence skin hydration through their fragmentation of HA molecules, affecting their ability to retain moisture [34]. In contrast, the antioxidant system works to counter the detrimental effects of ROS, thereby limiting the appearance of photoaging signs [7].

In terms of antioxidation, our study observed that cacao powder increased the activity of SOD and GPx in H_2_O_2_-treated KCs and reduced the expression of *NRF2* genes in UVB-irradiated HDFs. These outcomes substantiated the clinical improvements in skin wrinkles and hydration due to cacao powder in our study, which are the eventual consequences of oxidative stress. In the antioxidant system, NRF2 plays the role of a coordinator of multiple responses to oxidative stress. Among the multitude of reactions that NRF2 mediates, the catabolism of ROS, such as superoxide and peroxides, through SOD and GPx is one of the major mechanisms central to the antioxidant system [7]. As validated in prior studies, UV radiation led to the activation of NRF2, thereby limiting ECM degradation and protecting the skin against ROS [35,36]. Our results suggest that cacao powder can also collaboratively regulate antioxidant responses with NRF2, therefore reducing the need for its activation. The effects of cacao powder on the antioxidant system in our study serve as supporting evidence for in vivo studies. Lee et al. reported that cacao phenol extract demonstrated free radical-scavenging activity in a mouse skin model and reduced the generation of superoxide anions in cultured human promyelocytic leukemia cells [22]. Another mouse model study showed that a diet mixed with 16% cacao powder ameliorated the oxidative stress signals and increased antioxidant expression in rat brains after 8 weeks [18]. As a whole, our study contributes more evidence for the protective properties of cacao against ROS-induced damage, particularly in the skin.

The strengths of our study include a simultaneous assessment of skin hydration and wrinkles in a clinical trial and in vitro experiments, contributing both clinical and molecular evidence for the anti-photoaging functions of cacao powder on the skin. Furthermore, our study had a 12-week course of cacao powder supplementation for participants, limiting the impact of seasonal fluctuations in skin hydration status. Additionally, the placebo group and test group did not have any difference in some confounding factors that can affect the skin condition of participants in our clinical study, such as diet and cosmetic use. On the other hand, our limitations include a lack of other clinical assessments, such as skin elasticity, density, and thickness. Future studies are warranted to further judge the full potential of cacao in preventing photoaging.

Taken together, our study demonstrated that cacao powder exhibits significant anti-photoaging properties, encompassing improvements in skin hydration, wrinkle prevention, and antioxidant defenses. To be specific, cacao powder clinically enhanced skin hydration better than the placebo group. This was corroborated by in vitro findings where cacao powder treatment led to increased expression of HA and HAS-2 in KCs. Additionally, cacao powder administration clinically decreased the appearance of wrinkles more than the placebo group. This was supported by the increased expression of collagen type I and *SERPIN B6* genes while concurrently downregulating MMP-1 in UVB-irradiated HDFs after treatment with cacao powder. Finally, cacao powder exhibited potent antioxidant properties, which are crucial for countering oxidative stress induced by UV radiation. This was evidenced by the upregulation of antioxidant enzymes such as SOD and GPx in H_2_O_2_-treated KC, alongside a reduction in the expression of *NRF2* genes in UVB-irradiated HDFs. From these findings, we suggest that cacao powder has the potential to be developed as a comprehensive anti-photoaging product. Future studies are necessary to ascertain the long-term efficacy and reverse reaction of cacao powder, as well as the potency of other cacao-based formulations, such as topical or injecting agents.

## 5. Conclusions

Clinical and laboratory testing showed that cacao powder possesses anti-aging properties for the skin, from improving hydration and reducing wrinkles to defending against oxidative stress. Oral supplementation of cacao powder could be a promising prospect as a novel and accessible method of skin rejuvenation.

## Data Availability

The data that support the findings of this study are available from the corresponding author upon reasonable request.

## Supplementary Materials

The following supporting information can be downloaded at: https://www.mdpi.com/article/10.3390/cimb46110746/s1, Figure S1. Cell viability according to cacao powder concentration on HDF and KC. HDF, human dermal fibroblast; KC, keratinocyte; *** *p* < 0.005, independent samples *t*-test. *n* = 3, The error bars are based on standard deviation; Figure S2. Flowchart of the procedure to recruit, screen, and randomize participants; Table S1: The ingredients of placebo and test products; Table S2: The assessment table of participants’ skin wrinkles grade; Table S3: Demographic information of participants; Table S4: Nutritional analysis of participants.
