# Supplementary material for: The Role of Cacao Powder in Enhancing Skin Moisture and Reducing Wrinkles: A 12-Week Clinical Trial and In Vitro Study
Source: Curr Issues Mol Biol. 2024 Nov 6;46(11):12574–87. doi: 10.3390/cimb46110746 (PMC11592858; doi:10.3390/cimb46110746)
Supplement: Supplementary file 1 [file cimb-46-00746-s001.zip › cimb-3259513-supplementary.pdf]

Supplementary Materials

Figure S1. Cell viability of according to cacao powder concentration on HDF and KC. HDF, human dermal fibroblast; KC, keratinocyte; ***$p < 0.005$, independent samples *t*-test. N = 3, The error bars are based on standard deviation

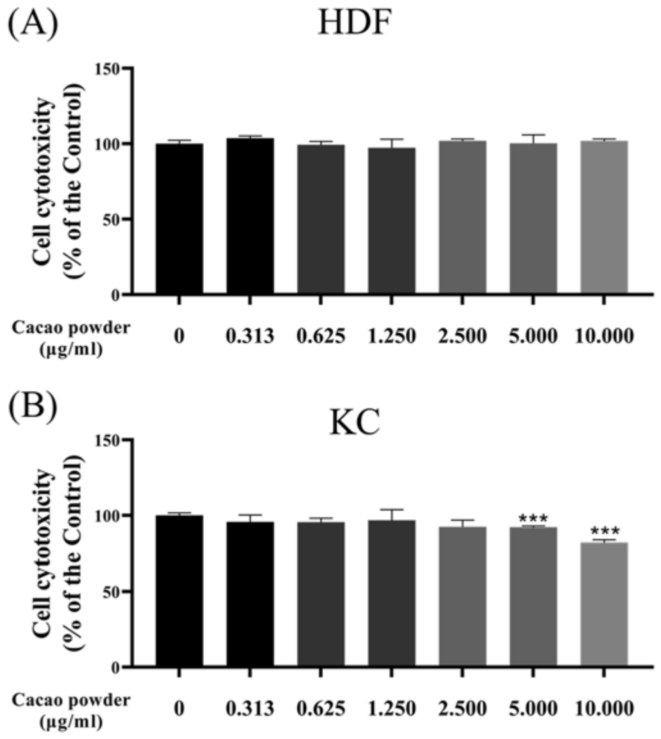

Figure S2. Flowchart of the procedure to recruit, screen, and randomize participants.

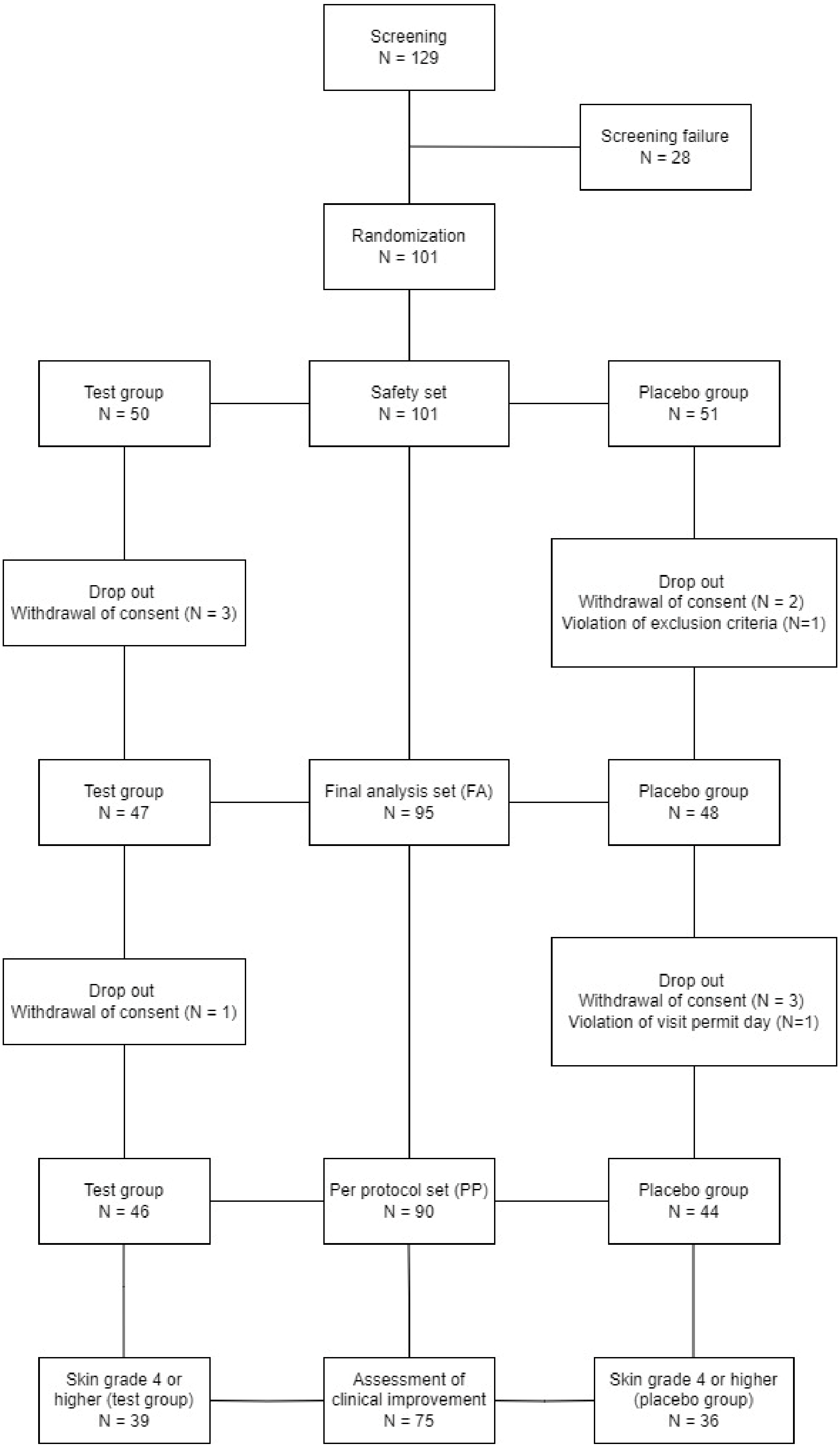

Table S1. The ingredients of placebo and test products.

Placebo product		Test product	
Ingredient	Ratio (%)	Ingredient	Ratio (%)
Non-glutinous Rice Powder	40.366	Cacao Powder	95.24
Sticky Rice Powder	18.348	Hydroxypropyl Methylcellulose	2.38
Corn Starch	16.417	Glutinous Rice Powder	1.33
Starch Syrup	14.486	Caramel Color	0.6
Caramel Color	4.463	Glycerin Fatty Acid Ester	0.45
Maltodextrin	2.897		
Hydroxypropyl Methylcellulose	2.38		
Glycerin Fatty Acid Ester	0.45		
Chocolate Flavor	0.193		

Participants consumed 97 pills from a single packet to achieve a total intake of 4 g of cacao powder per day, equating to 4.2 g including other ingredients.

Table S2. The assessment table of participants' skin wrinkles grade.

Grade	Description
0	No wrinkles and skin roughness is delicate
1	Fine wrinkles are beginning to appear
2	Slight formation of fine wrinkles
3	Numerous fine wrinkles progressing to shallow wrinkles
4	Slight formation of shallow wrinkles
5	Shallow wrinkles have become more pronounced, but no deep wrinkles
6	Shallow wrinkles are progressing to deep wrinkles
7	Slight formation of deep wrinkles
8	Numerous deep wrinkles are present
9	Wrinkles are very deep and numerous

Table S3. Demographic information of participants

		Test group N=44	Placebo group N=48
Gender n (%)			
p = 0.7195 (C)	Male	7 (15.91)	9 (18.75)
	Female	37 (84.09)	39 (81.25)
Age			
p = 0.2467 (W)	Mean±SD	44.64±6.52	46.25±6.86
Prior facial procedures (botox, filler, etc.) within the last 6 months n (%)			
	Yes	0 (0.00)	0 (0.00)
	No	44 (100.00)	48 (100.00)
Prior skin care (laser treatment on the face, peeling, other skin care, etc.) within 1 month (30 days) n (%)			
	Yes	0 (0.00)	0 (0.00)
	No	44 (100.00)	48 (100.00)
Alcohol drinking n (%)			
p = 0.3008 (C)	No	20 (45.45)	27 (56.25)
	Yes	24 (54.55)	21 (43.75)
Smoking n (%)			
p = 0.18894 (F)	No	40 (90.91)	47 (97.92)
	Quit smoking for more than 1 year	4 (9.09)	1 (2.08)
	Quit smoking for less than 1 year	0 (0.00)	0 (0.00)
	Still smoking	0 (0.00)	0 (0.00)
Outdoor activity time n (%)			
p = 0.6632 (F)	Less than 3 hours/day	36 (81.82)	35 (72.92)
	From 3 to less than 5 hours/day	5 (11.36)	8 (16.67)

	5 hours or more/day	3 (6.82)	5 (10.42)
Sunscreen frequency n (%)			
p = 0.7968 (C)	0 times/week	11 (25.00)	14 (29.17)
	1~2 times/week	7 (15.91)	5 (10.42)
	3~4 times/week	10 (22.73)	9 (18.75)
	5 times or more/week	16 (36.36)	20 (41.67)

- Compared between groups; p-value for Chi-square test(C) or Fisher's exact test(F), and Wilcoxon rank sum test(W)

Table S4. Nutritional analysis of participants

Table S4.1. Energy (kcal)

		Test group N=44		Placebo group N=48
Visit 2 (Baseline)	n	44		48
	Mean±SD	1,426.86±464.52		1,403.77±427.13
	Median	1,370.80		1,365.84
	Min, Max	411.50, 2,984.57		633.84, 2,622.82
	p-value [2]		0.8044(T)	
Visit 3 (6 weeks)	n	44		48
	Mean±SD	1,631.41±434.16		1,845.79±842.92
	Median	1,591.73		1,671.61
	Min, Max	746.11, 2,476.44		589.95, 6,207.88
Change from baseline	n	44		48
	Mean±SD	204.55±446.98		442.02±832.88
	Median	208.75		450.23
	Min, Max	-595.63, 1,302.41		-1,132.81, 4,488.86
	p-value [1]	0.0041		0.0006
	p-value [2]		0.0780(W)	
Visit 4 (12 weeks)	n	44		48
	Mean±SD	1,566.25±356.41		1,619.48±516.65
	Median	1,591.87		1,542.86
	Min, Max	726.29, 2,252.84		474.77, 3,258.64
Change from baseline	n	44		48
	Mean±SD	139.39±501.08		215.71±526.85
	Median	174.65		201.12
	Min, Max	-1,632.27, 1,166.92		-739.35, 1,845.93
	p-value [1]	0.0719		0.0067
	p-value [2]		0.4792(T)	

[1] Compared within groups; p-value for Paired t-test

[2] Compared between groups; p-value for Two sample t-test(T) or Wilcoxon rank sum test(W)

Table S4.2. Carbohydrate (g)

		Test group N=44		Placebo group N=48
Visit 2 (Baseline)	n	44		48
	Mean±SD	188.33±65.36		189.36±64.26
	Median	184.21		190.56
	Min, Max	46.25, 387.62		57.37, 306.35
	p-value [2]		0.9397(T)	
Visit 3 (6 weeks)	n	44		48
	Mean±SD	210.13±57.03		232.73±84.44
	Median	198.23		220.21
	Min, Max	82.08, 320.85		90.99, 488.44
Change from baseline	n	44		48
	Mean±SD	21.80±72.12		43.38±84.18
	Median	22.60		50.94
	Min, Max	-132.79, 157.39		-95.83, 277.60
	p-value [1]	0.0513		0.0008
	p-value [2]		0.1921(T)	
Visit 4 (12 weeks)	n	44		48
	Mean±SD	207.11±56.20		203.92±65.13
	Median	196.25		196.97
	Min, Max	104.63, 330.38		46.55, 389.73
Change from baseline	n	44		48
	Mean±SD	18.78±75.87		14.57±68.18
	Median	20.28		31.94
	Min, Max	-178.77, 165.62		-133.91, 136.64
	p-value [1]	0.1078		0.1455
	p-value [2]		0.7814(W)	

[1] Compared within groups; p-value for Paired t-test

[2] Compared between groups; p-value for Two sample t-test(T) or Wilcoxon rank sum test(W)

Table S4.3. Lipid (g)

		Test group<br>N=44		Placebo group<br>N=48
Visit 2 (Baseline)	n	44		48
	Mean±SD	47.36±34.30		42.54±24.05
	Median	42.33		40.36
	Min, Max	4.76, 233.07		10.05, 123.71
	p-value [2]		0.4140(W)	
Visit 3 (6 weeks)	n	44		48
	Mean±SD	54.84±29.30		66.49±48.03
	Median	52.62		55.69
	Min, Max	18.51, 184.17		11.96, 281.36
Change from baseline	n	44		48
	Mean±SD	7.47±25.37		23.95±51.89
	Median	7.65		21.25
	Min, Max	-48.90, 76.07		-79.53, 239.00
	p-value [1]	0.0572		0.0025
	p-value [2]		0.0307(W)	
Visit 4 (12 weeks)	n	44		48
	Mean±SD	50.23±19.26		55.90±30.96
	Median	48.94		51.57
	Min, Max	15.26, 104.21		14.90, 186.47
Change from baseline	n	44		48
	Mean±SD	2.86±37.04		13.36±34.05
	Median	10.57		10.94
	Min, Max	-194.16, 46.11		-61.19, 166.30
	p-value [1]	0.6106		0.0091
	p-value [2]		0.4414(W)	

[1] Compared within groups; p-value for Paired t-test

[2] Compared between groups; p-value for Two sample t-test(T) or Wilcoxon rank sum test(W)

Table S4.4. Protein (g)

		Test group N=44		Placebo group N=48
Visit 2 (Baseline)	n	44		48
	Mean±SD	59.00±25.87		58.79±20.77
	Median	56.54		58.59
	Min, Max	10.98, 148.83		11.14, 99.27
	p-value [2]		0.7046(W)	
Visit 3 (6 weeks)	n	44		48
	Mean±SD	70.69±25.16		74.51±49.24
	Median	70.69		67.96
	Min, Max	32.74, 165.63		20.12, 370.87
Change from baseline	n	44		48
	Mean±SD	11.68±33.70		15.72±48.24
	Median	4.24		11.90
	Min, Max	-70.26, 119.86		-42.32, 292.05
	p-value [1]	0.0264		0.0286
	p-value [2]		1.0000(W)	
Visit 4 (12 weeks)	n	44		48
	Mean±SD	64.44±17.27		70.82±29.74
	Median	61.39		66.97
	Min, Max	21.07, 101.45		22.06, 193.90
Change from baseline	n	44		48
	Mean±SD	5.44±27.94		12.03±27.40
	Median	8.28		8.91
	Min, Max	-88.94, 67.22		-33.18, 115.08
	p-value [1]	0.2039		0.0038
	p-value [2]		0.5656(W)	

[1] Compared within groups; p-value for Paired t-test

[2] Compared between groups; p-value for Two sample t-test(T) or Wilcoxon rank sum test(W)

Table S4.5. Dietary fiber (g)

		Test group N=44		Placebo group N=48
Visit 2 (Baseline)	n	44		48
	Mean±SD	13.81±4.87		15.57±6.70
	Median	13.13		15.00
	Min, Max	5.96, 24.86		5.07, 31.59
	p-value [2]		0.1490(T)	
Visit 3 (6 weeks)	n	44		48
	Mean±SD	17.35±5.22		18.41±8.04
	Median	17.52		16.86
	Min, Max	6.09, 32.79		8.08, 44.30
Change from baseline	n	44		48
	Mean±SD	3.54±6.81		2.84±7.45
	Median	4.61		3.21
	Min, Max	-13.78, 15.86		-13.57, 25.94
	p-value [1]	0.0013		0.0112
	p-value [2]		0.6384(T)	
Visit 4 (12 weeks)	n	44		48
	Mean±SD	15.80±5.30		17.38±7.06
	Median	16.23		15.63
	Min, Max	7.44, 34.61		5.93, 40.65
Change from baseline	n	44		48
	Mean±SD	1.99±6.94		1.80±7.42
	Median	1.53		1.86
	Min, Max	-15.02, 27.39		-16.98, 26.19
	p-value [1]	0.0636		0.0988
	p-value [2]		0.9408(W)	

[1] Compared within groups; p-value for Paired t-test

[2] Compared between groups; p-value for Two sample t-test(T) or Wilcoxon rank sum test(W)

Table S4.6. Moisture (g)

		Test group N=44		Placebo group N=48
Visit 2 (Baseline)	n	44		48
	Mean±SD	668.60±300.25		711.02±301.06
	Median	618.90		703.99
	Min, Max	47.33, 1,603.82		146.87, 1,730.62
	p-value [2]		0.5008(T)	
Visit 3 (6 weeks)	n	44		48
	Mean±SD	817.91±228.15		888.72±404.87
	Median	786.75		840.42
	Min, Max	423.70, 1,384.08		386.93, 2,513.80
Change from baseline	n	44		48
	Mean±SD	149.30±324.38		177.70±372.94
	Median	138.15		189.33
	Min, Max	-577.03, 785.51		-618.97, 1,515.90
	p-value [1]	0.0039		0.0018
	p-value [2]		0.9470(W)	
Visit 4 (12 weeks)	n	44		48
	Mean±SD	792.23±221.05		839.17±334.12
	Median	749.54		803.58
	Min, Max	349.78, 1,222.57		332.38, 2,398.56
Change from baseline	n	44		48
	Mean±SD	123.63±306.26		128.15±251.96
	Median	108.11		136.76
	Min, Max	-472.69, 942.75		-393.38, 667.94
	p-value [1]	0.0105		0.0010
	p-value [2]		0.9384(T)	

[1] Compared within groups; p-value for Paired t-test

[2] Compared between groups; p-value for Two sample t-test(T) or Wilcoxon rank sum test(W)

Table S4.7. Vitamin A (μg RE)

		Test group N=44		Placebo group N=48
Visit 2 (Baseline)	n	44		48
	Mean±SD	408.71±259.15		422.67±336.13
	Median	342.94		287.34
	Min, Max	28.47, 1,260.08		28.93, 1,390.57
	p-value [2]		0.4185(W)	
Visit 3 (6 weeks)	n	44		48
	Mean±SD	496.43±293.09		600.69±440.05
	Median	431.98		497.42
	Min, Max	113.77, 1,915.48		108.23, 1,982.55
Change from baseline	n	44		48
	Mean±SD	87.72±446.78		178.02±453.36
	Median	51.46		133.92
	Min, Max	-860.24, 1,887.02		-876.25, 1,698.56
	p-value [1]	0.1997		0.0091
	p-value [2]		0.2212(W)	
Visit 4 (12 weeks)	n	44		48
	Mean±SD	507.89±241.94		561.30±398.76
	Median	439.17		466.86
	Min, Max	129.03, 1,111.74		70.75, 2,112.99
Change from baseline	n	44		48
	Mean±SD	99.18±371.21		138.63±470.18
	Median	105.73		104.24
	Min, Max	-1,012.85, 1,083.27		-853.92, 1,964.25
	p-value [1]	0.0834		0.0467
	p-value [2]		0.7397(W)	

[1] Compared within groups; p-value for Paired t-test

[2] Compared between groups; p-value for Two sample t-test(T) or Wilcoxon rank sum test(W)

Table S4.8. Niacin (mg)

		Test group<br>N=44		Placebo group<br>N=48
Visit 2 (Baseline)	n	44		48
	Mean±SD	10.86±7.22		9.37±4.86
	Median	9.74		8.43
	Min, Max	1.19, 40.75		1.92, 22.16
	p-value [2]		0.3835(W)	
Visit 3 (6 weeks)	n	44		48
	Mean±SD	11.31±4.35		13.37±8.28
	Median	10.35		12.19
		Min, Max		3.49, 57.71
Change from baseline	n	44		48
	Mean±SD	0.45±7.13		4.00±7.13
	Median	1.25		3.87
	Min, Max	-23.04, 13.47		-7.17, 40.21
	p-value [1]	0.6793		0.0003
	p-value [2]		0.0313(W)	
Visit 4 (12 weeks)	n	44		48
	Mean±SD	10.53±3.65		11.94±6.17
	Median	9.75		10.90
	Min, Max	4.34, 22.09		3.67, 37.44
Change from baseline	n	44		48
	Mean±SD	-0.33±7.75		2.57±6.28
	Median	0.25		2.59
	Min, Max	-29.42, 13.56		-13.52, 30.05
	p-value [1]	0.7764		0.0067
	p-value [2]		0.0530(W)	

[1] Compared within groups; p-value for Paired t-test

[2] Compared between groups; p-value for Two sample t-test(T) or Wilcoxon rank sum test(W)

Table S4.9. Vitamin C (mg)

		Test group N=44		Placebo group N=48
Visit 2 (Baseline)	n	44		48
	Mean±SD	46.85±55.38		50.57±55.04
	Median	24.01		28.42
	Min, Max	0.00, 237.54		5.30, 281.00
	p-value [2]		0.4276(W)	
Visit 3 (6 weeks)	n	44		48
	Mean±SD	84.22±71.74		72.03±77.03
	Median	63.02		42.45
	Min, Max	10.67, 313.65		8.95, 461.06
Change from baseline	n	44		48
	Mean±SD	37.37±86.63		21.46±84.36
	Median	20.78		18.91
	Min, Max	-115.00, 299.77		-224.07, 338.49
	p-value [1]	0.0065		0.0845
	p-value [2]		0.5763(W)	
Visit 4 (12 weeks)	n	44		48
	Mean±SD	60.66±44.14		60.87±42.61
	Median	46.99		51.22
	Min, Max	9.14, 172.53		8.68, 195.58
Change from baseline	n	44		48
	Mean±SD	13.81±68.19		10.30±74.77
	Median	19.19		13.02
	Min, Max	-183.95, 127.79		-240.35, 184.14
	p-value [1]	0.1862		0.3446
	p-value [2]		0.6588(W)	

[1] Compared within groups; p-value for Paired t-test

[2] Compared between groups; p-value for Two sample t-test(T) or Wilcoxon rank sum test(W)

Table S4.10. Vitamin D (μg)

		Test group N=44		Placebo group N=48
Visit 2 (Baseline)	n	44		48
	Mean±SD	1.18±1.70		1.41±2.54
	Median	0.69		0.63
	Min, Max	0.00, 8.81		0.00, 15.72
	p-value [2]		0.9283(W)	
Visit 3 (6 weeks)	n	44		48
	Mean±SD	2.17±3.06		1.64±1.64
	Median	1.10		1.09
	Min, Max	0.02, 13.32		0.00, 7.75
Change from baseline	n	44		48
	Mean±SD	0.99±3.71		0.23±2.87
	Median	0.60		0.29
	Min, Max	-8.17, 12.92		-14.80, 4.56
	p-value [1]	0.0848		0.5866
	p-value [2]		0.6475(W)	
Visit 4 (12 weeks)	n	44		48
	Mean±SD	1.28±0.88		1.49±1.43
	Median	1.27		0.97
	Min, Max	0.09, 4.54		0.05, 6.70
Change from baseline	n	44		48
	Mean±SD	0.10±1.84		0.07±2.82
	Median	0.26		0.35
	Min, Max	-8.16, 4.54		-14.77, 4.36
	p-value [1]	0.7092		0.8567
	p-value [2]		0.5709(W)	

[1] Compared within groups; p-value for Paired t-test

[2] Compared between groups; p-value for Two sample t-test(T) or Wilcoxon rank sum test(W)

Table S4.11. Vitamin E (mg)

		Test group N=44		Placebo group N=48
Visit 2 (Baseline)	n	44		48
	Mean±SD	9.98±13.01		9.34±5.67
	Median	6.81		8.82
	Min, Max	0.98, 85.32		1.50, 30.75
	p-value [2]		0.2967(W)	
Visit 3 (6 weeks)	n	44		48
	Mean±SD	11.58±8.70		14.34±13.70
	Median	9.85		11.87
	Min, Max	2.67, 60.92		3.03, 92.72
Change from baseline	n	44		48
	Mean±SD	1.59±8.64		5.00±15.49
	Median	3.77		3.51
	Min, Max	-24.41, 20.67		-26.59, 91.22
	p-value [1]	0.2280		0.0302
	p-value [2]		0.6307(W)	
Visit 4 (12 weeks)	n	44		48
	Mean±SD	10.51±4.88		13.33±12.14
	Median	9.46		10.72
	Min, Max	2.13, 22.46		4.01, 64.42
Change from baseline	n	44		48
	Mean±SD	0.53±14.23		3.99±11.70
	Median	3.09		2.30
	Min, Max	-77.19, 16.76		-16.07, 58.56
	p-value [1]	0.8074		0.0224
	p-value [2]		0.8789(W)	

[1] Compared within groups; p-value for Paired t-test

[2] Compared between groups; p-value for Two sample t-test(T) or Wilcoxon rank sum test(W)

Table S4.12. Zinc (mg)

		Test group<br>N=44		Placebo group<br>N=48
Visit 2 (Baseline)	n	44		48
	Mean±SD	6.19±3.02		6.50±3.39
	Median	6.15		5.95
	Min, Max	0.75, 14.49		0.64, 17.22
	p-value [2]		0.9782(W)	
Visit 3 (6 weeks)	n	44		48
	Mean±SD	7.47±2.66		8.24±4.92
	Median	6.80		7.20
	Min, Max	2.98, 14.40		2.56, 33.70
Change from baseline	n	44		48
	Mean±SD	1.28±3.69		1.74±5.33
	Median	0.60		1.81
	Min, Max	-5.81, 10.14		-6.72, 25.64
	p-value [1]	0.0261		0.0280
	p-value [2]		0.7221(W)	
Visit 4 (12 weeks)	n	44		48
	Mean±SD	6.85±2.35		7.22±3.10
	Median	6.98		6.65
	Min, Max	1.73, 12.58		2.08, 17.32
Change from baseline	n	44		48
	Mean±SD	0.67±3.72		0.73±3.70
	Median	0.58		1.38
	Min, Max	-9.74, 7.67		-10.00, 7.17
	p-value [1]	0.2414		0.1811
	p-value [2]		0.5499(W)	

[1] Compared within groups; p-value for Paired t-test

[2] Compared between groups; p-value for Two sample t-test(T) or Wilcoxon rank sum test(W)

Table S4.13. Copper (mg)

		Test group N=44	Placebo group N=48
Visit 2 (Baseline)	n	44	48
	Mean±SD	441.21±241.52	571.15±392.20
	Median	406.05	426.86
	Min, Max	94.60, 992.33	77.00, 1,788.67
	p-value [2]	0.1801(W)	
Visit 3 (6 weeks)	n	44	48
	Mean±SD	587.65±226.97	657.34±354.42
	Median	550.52	586.49
	Min, Max	210.49, 1,108.65	181.63, 1,666.72
Change from baseline	n	44	48
	Mean±SD	146.45±355.24	86.19±471.75
	Median	145.27	184.91
	Min, Max	-568.90, 1,014.05	-1,235.77, 940.44
	p-value [1]	0.0090	0.2118
	p-value [2]	0.8604(W)	
Visit 4 (12 weeks)	n	44	48
	Mean±SD	539.15±220.35	622.71±407.40
	Median	495.05	536.43
	Min, Max	129.28, 1,297.91	154.82, 2,880.08
Change from baseline	n	44	48
	Mean±SD	97.95±295.82	51.56±406.35
	Median	109.19	93.88
	Min, Max	-548.05, 810.62	-1,284.65, 1,091.41
	p-value [1]	0.0335	0.3838
	p-value [2]	0.9036(W)	

[1] Compared within groups; p-value for Paired t-test

[2] Compared between groups; p-value for Two sample t-test(T) or Wilcoxon rank sum test(W)

Table S4.14. Selenium (μg)

		Test group<br>N=44		Placebo group<br>N=48
Visit 2 (Baseline)	n	44		48
	Mean±SD	62.83±31.42		65.81±33.33
	Median	57.45		64.24
	Min, Max	3.07, 136.04		7.20, 183.13
	p-value [2]		0.6609(T)	
Visit 3 (6 weeks)	n	44		48
	Mean±SD	82.95±37.46		73.88±32.99
	Median	73.80		64.52
	Min, Max	32.99, 200.42		19.99, 216.68
Change from baseline	n	44		48
	Mean±SD	20.12±45.13		8.07±39.63
	Median	14.22		5.08
	Min, Max	-65.44, 176.76		-107.64, 131.35
	p-value [1]	0.0050		0.1647
	p-value [2]		0.2931(W)	
Visit 4 (12 weeks)	n	44		48
	Mean±SD	78.58±26.70		78.36±41.27
	Median	75.65		67.83
	Min, Max	22.79, 145.47		20.20, 227.40
Change from baseline	n	44		48
	Mean±SD	15.75±37.25		12.55±40.50
	Median	15.77		11.71
	Min, Max	-81.76, 96.96		-76.78, 100.71
	p-value [1]	0.0075		0.0370
	p-value [2]		0.6946(T)	

[1] Compared within groups; p-value for Paired t-test

[2] Compared between groups; p-value for Two sample t-test(T) or Wilcoxon rank sum test(W)
